# Potential Treatment Options for COVID-19: A Comprehensive Review of Global Pharmacological Development Efforts

**DOI:** 10.7759/cureus.8845

**Published:** 2020-06-26

**Authors:** Karun Neupane, Zahoor Ahmed, Hira Pervez, Rabia Ashraf, Aneela Majeed

**Affiliations:** 1 Internal Medicine, Manipal College of Medical Sciences, Pokhara, NPL; 2 Internal Medicine, King Edward Medical University, Mayo Hospital, Lahore, PAK; 3 Internal Medicine/Cardiology, Dow University of Health Sciences, Karachi, PAK; 4 Internal Medicine, King Edward Medical University, Lahore, PAK; 5 Infectious Diseases, Cleveland Clinic, Cleveland, USA

**Keywords:** sars-cov-2, covid-19, hydroxychloroquine, remdesivir, coronavirus, treatment, therapeutics

## Abstract

Coronavirus disease-2019 (COVID-19), first reported in China during December of 2019, is caused by the severe acute respiratory syndrome coronavirus 2 (SARS-CoV-2). Infection later spread very rapidly around the globe with over 8,708,008 cases reported, including more than 461,715 deaths reported across at least 216 countries by June 20, 2020. It was declared as a global pandemic by the World Health Organization (WHO) on March 11, 2020. With the rapidly increasing number of positive cases and deaths, there is a dire need for effective treatment. An urgent unmet need led to the planning and opening of multiple drug development trials for treatment and vaccine development. In this article, we have compiled comprehensive data on many candidate drugs such as remdesivir, favipiravir, ribavirin, umifenovir, arbidol, lopinavir, ritonavir, baricitinib, hydroxychloroquine, nitazoxanide, azithromycin, baloxavir, oseltamivir, losartan, and tocilizumab. We have tabulated available data on various clinical trials testing various aspects of COVID-19 therapeutics.

## Introduction and background

Severe acute respiratory syndrome coronavirus 2 (SARS-COV-2), the causative agent of COVID-19 (coronavirus disease-2019), first originated from Wuhan in the Hubei province of China during late December of 2019. It started as an outbreak which led to an epidemic with 44,672 confirmed cases in China by February 14, 2020, with 2.3% reported mortality rate, which was comparatively lower than the previously known epidemics caused by human coronaviruses (Severe Acute Respiratory Syndrome Coronavirus [SARS-CoV] and the Middle East Respiratory Syndrome Coronavirus [MERS-CoV]) in 2003 and 2012, respectively [1]. It rapidly spread outside of China to the rest of the world, mainly through human-to-human transmission by respiratory droplets or possibly through the fecal-oral route, and consequently, the World Health Organization (WHO) declared COVID-19 as a global pandemic on March 11, 2020 [2]. The infection presents most commonly as fever, dry cough, shortness of breath, sore throat, and diarrhea with severe respiratory involvement in patients with advanced age (over age 80). The overall case fatality rate in this age group is about 14.3% [3]. The severity of this disease is characterized by severe pneumonia, respiratory failure requiring mechanical ventilation, sepsis, myocardial injury, multi-organ failure, and mortality increases in patients having underlying comorbidities such as cardiovascular disease, diabetes, and chronic respiratory disease [4]. Given the rapid spread of the virus, researchers across multiple nations have dedicated themselves to better understand the virus and disease pathophysiology and develop effective drugs and preventive vaccines.

While new treatment options are being sought out, one of the main areas of focus has been trying to repurpose the existing drugs to fight against COVID-19. Several drugs are being tested in the trials, and the United States Food and Drugs Administration (FDA) has given Emergency Use Authorization (EUA) for remdesivir to treat COVID-19 patients on May 1, 2020 [5].

## Review

The clinical management of COVID-19 patients is focused on alleviating clinical symptoms by general and symptom-specific supportive care [6]. Potential therapeutic options against COVID-19 include molecules binding to the virus, inhibitors that can target specific enzymes involved in viral replication or viral transcription. Small-molecule inhibitors can target helicase, essential proteases, or other proteins of the virus, and host cell protease inhibitor [7]. Based on the sequencing of the entire genome and the information coupled with protein structure modeling, the research community has been able to rapidly respond with a proposed list of antiviral agents with potential therapeutic efficacy in the treatment of COVID-19. The investigational drugs together with their potential mechanisms of actions and other specific characteristics are summarized in Table 1.

**Table 1:** Drugs repurposed for the treatment of COVID-19 under clinical trials RNA, Ribonucleic acid; mRNA, Messenger RNA; HIV, Human immunodeficiency virus; JAK, Janus kinase; ACE2, Angiotensin-converting enzyme 2; SARS-CoV-2, Severe acute respiratory syndrome coronavirus 2; COVID-19, Coronavirus disease 2019

Drug	Mechanism of Action (MOA)	Disease indication
Remdesivir	Metabolizes into its active form GS-441524, interferes with RNA polymerase and decreases viral RNA production; may inhibit viral nucleotide synthesis to stop viral replication	Ebola virus infection
Corticosteroids	Binds with cytoplasmic receptors and alters the transcription of mRNA and reduce the production of inflammatory mediators responsible for the disease.	Hyperglycemia, adrenal suppression
Favipiravir (Favilavir)	RNA replicase; a purine nucleoside that acts as an alternate substrate leading to lethal RNA transversion mutations, producing a nonviable viral phenotype	Viral infections
Ribavirin	Metabolized to purine RNA to stop viral synthesis and viral mRNA capping	RSV infection, hepatitis C
Umifenovir (Arbidol)	Inhibits membrane fusion and prevents contact between virus and host cell; an inhibitor that may disrupt the binding of viral envelope protein to host cells and thus may prevent viral entry to the target cell	Influenza
Lopinavir (LPV)	Viral protease inhibitor that may inhibit the viral replication	HIV infection
Ritonavir (RTV)	Pharmacokinetic profile enhancer of other protease inhibitors	
Baricitinib	Inhibitor of Janus kinase (JAK) that may interfere with the inflammatory processes	Rheumatoid arthritis
Hydroxychloroquine	Chloroquine analog, lysosomotropic agent; can elevate endosomal pH and interfere with ACE2 glycosylation	Malaria
Nitazoxanide	Prototype member of the thiazolides which has antiviral activity and inhibits viral protein expression	Various helminthic, protozoal, and viral infection
Azithromycin	Interferes with protein synthesis by inhibiting the translation of mRNA; possible mechanism in COVID-19 due to enhancement of the action of hydroxychloroquine	Bacterial infections
Baloxavir	An enzyme inhibitor targeting the activities of virus polymerase complexes	Influenza virus
Oseltamivir	A competitive inhibitor of neuraminidase; cleaves sialic acid which prevents the release of the new viral particle from the cell	Influenza virus
Tocilizumab (Actemra®)	May potentially combat cytokine release syndrome (CRS) symptoms (e.g., fever, organ failure, death) in severely ill patients	Rheumatoid arthritis, severe or life‐threatening chimeric antigen receptor (CAR) T cell‐induced cytokine release syndrome (CRS)
Avastin (Bevacizumab	Vascular endothelial growth factor inhibitor that binds to receptors on endothelial cells to help drive angiogenesis	Certain types of cancer
Losartan	Higher angiotensin-converting enzyme 2 expressions following long term treatment with angiotension1 receptor blocker losartan in SARS-CoV-2 infected patients may protect against acute lung injury	Hypertension and diabetic nephropathy

Antivirals

The 90% effective concentration (EC90) value of remdesivir against SARS-CoV-2 in Vero E6 cells was 1.76 μM, which suggests that its working concentration is likely to be achieved in non-human primates [8]. Remdesivir is available for the treatment of ebola virus disease in humans and has shown promising results in animal models for MERS-CoV and SARS-CoV. Therapeutic remdesivir treatment in MERS-CoV inoculated rhesus macaques resulted in the reduction in clinical signs, virus replication, and the absence of lung lesions in 2/6 remdesivir-treated animals along with the reduction in lesion severity in three additional animals. MERS-CoV has a close resemblance to SARS-CoV-2 and the drug is being studied for the treatment of COVID-19 in China and the United States [9]. Results from a recent open-labeled, nonrandomized trial of remdesivir in 53 COVID-19 patients showed that after up to ten days of compassionate use of remdesivir, 68% of the cohort showed symptoms improvement with only 13% mortality rate at a median follow up of 18 days. It is noteworthy to mention that 34 (64%) patients were already on invasive ventilation before the initiation of the treatment, out of which 20 patients were weaned off the invasive ventilation successfully following the treatment. The adverse effects with remdesivir ranged widely from increased liver enzymes (23%), diarrhea (9%), rash (8%) to more severe conditions being multiple organ failure (6%), septic shock (4%), hypotension (8%) and acute kidney injury (6%) [10]. In a randomized controlled clinical trial of 1063 patients conducted by the National Institute of Allergy and Infectious Disease (NIAID), remdesivir has shown the efficacy in the early results against advanced COVID-19 (NCT04280705). Patients were randomized into the remdesivir group and placebo group. It was observed that remdesivir was better from the perspective of primary endpoint and time to recovery defined as being well enough for hospital discharge or returning to the normal activity level. Preliminary results of this trial showed 31% faster time to recovery in those patients who received compassionate use of remdesivir as compared to placebo patients (*p* < 0.001). The median time to recovery was 11 days for remdesivir group compared with 15 days for placebo group. Results were also significant in terms of survival benefit, with 8% mortality in remdesivir group as compared to 11.6% in placebo group (*p* = 0.059) These results support the use of remdesivir for the patients who are hospitalized with COVID-19 and require supplemental oxygen therapy [11].

The American Center for Disease Control (CDC) in a public document on its website, showed that three patients were treated with remdesivir via compassionate use protocol among the first 12 patients confirmed to have COVID-19 in the United States. All patients reportedly have recovered, but the few side effects like transient gastrointestinal symptoms and aminotransferase elevation were reported, and the authors were unable to weigh the efficacy of remdesivir because of no comparator and confounding treatments, including the concomitant use of corticosteroids in one patient [12]. A recent update on the efficacy of RDV against COVID-19 by Gilead Sciences also reported that more than 1,700 patients have now been treated with RDV through the expanded access programs [13]. The ongoing clinical trials on remdesivir are given in Table 2.

**Table 2:** Characteristics of clinical trials studying remdesivir in patients with COVID-19

Clinical trial ID	Status	Study results	Conditions	Interventions	Locations
NCT04302766	Available	No results available	COVID-19	Remdesivir	USA
NCT04280705	Recruiting	No results available	COVID-19	Placebo and remdesivir	USA
NCT04315948	Recruiting	No results available	COVID-19	Remdesivir and other standard care	France
NCT04315948	Recruiting	No results available	COVID-19	Drug: remdesivir other: standard of care	France
NCT04314817	Recruiting	No results available	COVID-19	Drug: Any drug used to treat COVID-19	France
NCT04292899	Recruiting	No results available	COVID-19	Drug: remdesivir drug: standard of care	USA
NCT04292730	Recruiting	No results available	COVID-19	Drug: remdesivir drug: standard of care	USA
NCT04321616	Recruiting	No results available	COVID-19	Drug: remdesivir other: (standard of care) soc	Norway

The combination of lopinavir-ritonavir was used to treat adult hospitalized patients with severe COVID-19. However, treatment with lopinavir-ritonavir was not associated with a difference from standard care in the time to clinical improvement (hazard ratio for clinical improvement: 1.24; 95% confidence interval [CI]: 0.90 to 1.72). Mortality at 28 days was similar in the lopinavir-ritonavir group and the standard-care group (19.2% vs. 25.0%; difference, −5.8 percentage points; 95% CI, −17.3 to 5.7) [14]. Further studies are undergoing to test its efficacy. A fixed dose of the anti-HIV combination, lopinavir-ritonavir, is currently in clinical trials with arbidol or ribavirin but results are not yet available [15]. Favipiravir, which acts as an alternate substrate leading to lethal RNA transversion mutations producing a nonviable viral phenotype, has also been recently approved for clinical trials in treating the COVID-19 [16]. The clinical trials on lopinavir and ritonavir are given in Table 3.

**Table 3:** Characteristics of clinical trials studying lopinavir/ritonavir in patients with COVID-19

Clinical trial ID	Status	Study results	Conditions	Interventions	Locations
NCT04252885	Recruiting	No results available	COVID-19	Drug: Lopinavir and ritonavir tablets drug: Arbidol	China
NCT04307693	Recruiting	No results available	COVID-19	Drug: Lopinavir/ritonavir drug: Hydroxychloroquine sulfate	South Korea
NCT02845843	Recruiting	No results available	COVID-19	Drug: Combination of lopinavir /Ritonavir and Interferon beta-1b drug: Placebo	Saudi Arabia
NCT04276688	Completed	No results available	COVID-19	Drug: Lopinavir/ritonavir drug: Ribavirin drug: Interferon beta-1B	Hong Kong
NCT04321174	Recruiting	No results available	COVID-19	Drug: Lopinavir/ritonavir	Canada
NCT04251871	Recruiting	No results available	COVID-19	Drug: lopinavir/ritonavir	China
NCT04315948	Recruiting	No results available	COVID-19	Drug: remdesivir drug: lopinavir/ritonavir drug: hydroxychloroquine	France
NCT04351724	Recruiting	No results available	COVID-19	Drug: Chloroquine or hydroxychloroquine drug: Lopinavir/ritonavir	Austria
NCT04314817	Recruiting	No results available	COVID-19	Drug: Any drug used to treat Covid-19	France
NCT04328012	Recruiting	No results available	COVID-19	Drug: Lopinavir/ritonavir drug: Hydroxychloroquine sulfate drug: Placebos	United States
NCT04343768	Completed	No results available	COVID-19	Drug: Hydroxychloroquine drug: Lopinavir / ritonavir	Iran

Corticosteroids

The use of corticosteroids for the treatment of coronavirus infections is controversial. While it reduces the immunological damage, viral rebound, and adverse events remain the major concerns [17]. The use of corticosteroids in SARS patients has been associated with increased viral load, while side effects like avascular necrosis (seen in 9% of patients in a cohort in Hong Kong) and opportunistic infections like aspergillosis have been reported [18]. A significantly higher concentration of plasma viral RNA concentration was reported in the second and third weeks of illness in patients treated with hydrocortisone (n=9) as compared to those receiving placebo (n=7; p=0.023) [19]. However, in another study, better results were seen in patients of SARS-CoV (n = 190) treated with early high-dose steroids in combination with a quinolone (n=60) (zero deaths; meantime to discharge: 20.7 ± 4.6) vs other three treatment groups (n = 40,30,60) (two or more deaths in each group; meantime to discharge: 24.8 ± 5.5, 24.8 ± 6.4, 22.4 ± 5.9).

Preliminary reports from the RECOVERY (Randomised Evaluation of COVid-19 thERapY) trial (n=2104, received dexamethasone at a dose of 6mg once daily per oral/intravenous vs n=4321 patients received usual care alone) showed that dexamethasone showed improved survival in COVID-19 patients. Among the patients receiving usual care alone, the 28 days mortality in those requiring ventilation, requiring oxygen only, and in those not requiring any respiratory intervention was 41%, 25%, and 13%, respectively. Even though no benefit was seen among the patients not requiring respiratory support (rate ratio 1.22; 95% confidence interval [CI] 0.86-1.75; *p* = 0.14), but in the ventilated patients with COVID-19, dexamethasone decreased the deaths by one-third (rate ratio 0.65; 95% CI 0.48-0.88; *p *= 0.0003), and by one-fifth in the patients requiring oxygen only (rate ratio 0.80; 95% CI 0.67-0.96; *p *= 0.0021). These results indicated that for the prevention of one death, eight patients requiring ventilation needed to treat while this number was 25 in patients requiring oxygen only. These promising results mean the role of dexamethasone and possibly other steroids should be explored further for the treatment of COVID-19 patients [20]. The clinical trials on corticosteroids are given in Table 4.

**Table 4:** Characteristics of clinical trials studying corticosteroid in patients with COVID-19

Clinical trial ID	Status	Study results	Conditions	Interventions	Locations
NCT04355637	Recruiting	No results available	COVID-19	Drug: Inhaled budesonide	Spain
NCT04344288	Recruiting	No results available	COVID-19	Drug: Prednisone	France
NCT04327401	Recruiting	No results available	COVID-19	Drug: Dexamethasone	Brazil
NCT04355247	Recruiting	No results available	COVID-19	Methylprednisolone 80 milligrams/milliliters injectable suspension	Rico
NCT04343729	Recruiting	No results available	COVID-19	Methylprednisolone sodium succinate Placebo solution	Brazil
NCT02517489	Recruiting	No results available	COVID-19	Drug: Hydrocortisone drug: Placebo	France

Janus kinase inhibitor

Baricitinib has anti-inflammatory action and possible ability to inhibit viral entry into the cells [21]. It is a Janus kinase (JAK) inhibitor that binds to the cyclin G-associated kinase, a regulator of endocytosis. Baricitinib in therapeutic dose (either as 2mg or 4mg once daily) is enough to inhibit AP2-associated protein kinase 1 (AAK1), a regulator of the endocytosis process, which is implicated in the cellular viral entry process. Baricitinib can disrupt AAK1 causing the interruption of viral entry into the cell and intracellular assembly of virus particles, which makes it a potential drug for COVID-19 [22]. The clinical trials on JAKs are given in Table 5.

**Table 5:** Characteristics of clinical trials studying baricitinib in patients with COVID-19

Clinical trial ID	Status	Study Results	Conditions	Interventions	Locations
NCT04321993	Recruiting	No results available	COVID-19	Drug: Lopinavir/ritonavir drug: Hydroxychloroquine sulfate drug: Baricitinib	Canada
NCT04401579	Recruiting	No results available	COVID-19	Other: Placebo drug: Remdesivir drug: Baricitinib	USA

Antiprotozoal drugs

Nitazoxanide, an antiprotozoal drug, has antiviral potential against a wide range of viruses, including human and animal coronaviruses. It has also inhibited viral replication at a low-micro molar concentration in Vero E6 cells, with half-maximal effective concentration (EC50) of 2.12μM [14]. Ivermectin also acts as an anti-viral by inhibiting importin α/β1 and integrase proteins involved in viral replication [23]. A single treatment with ivermectin has shown to reduce SARS-CoV-2 RNA by ∼5000-fold at 48 hours in cell culture [24].

**Table 6:** Characteristics of a clinical trial studying nitazoxanide in patients with COVID-19

Clinical trial ID	Status	Study Results	Conditions	Interventions	Locations
NCT04341493	Recruiting	No results available	COVID-19	Nitazoxanide 500 Mg Hydroxychloroquine	Mexico

Interleukin receptor blocker

Plasma levels of Interleukin-2 (IL-2), IL-7, and IL-10 were found to be higher in more sick patients and those admitted in the intensive care unit [25]. Tocilizumab is a recombinant humanized monoclonal antibody specific for the interleukin-6 (IL-6) receptor and is used for the treatment of cytokine release syndrome. In a retrospective observational study involving twenty patients with severe or critical COVID-19, treatment with tocilizumab in addition to lopinavir, methylprednisolone, other symptom relievers, and oxygen therapy, resulted in body temperature of all the patients returning to normal on the first day of receiving tocilizumab and significant relief of clinical symptoms synchronously in the following days. After treatment, oxygen intake was lowered in 15/20 patients and one patient did not need oxygen therapy while CT scan lesions were absorbed in nineteen patients. No adverse events were reported during treatment with tocilizumab [26]. Ongoing clinical trials on tocilizumab are given in Table 7.

**Table 7:** Characteristics of clinical trials studying tocilizumab in patients with COVID-19

Clinical trial ID	Status	Study results	Conditions	Interventions	Locations
NCT04335071	Recruiting	No results available	COVID-19	Drug: Tocilizumab drug: Placebo	Switzerland
NCT04310228	Recruiting	No results available	COVID-19	Drug: Favipiravir combined with tocilizumab	China
NCT04359667	Recruiting	No results available	COVID-19	Drug: Tocilizumab 20 milligrams/milliliters intravenous solution [ACTEMRA]	Croatia
NCT04339712	Recruiting	No results available	COVID-19	Drug: Anakinra drug: Tocilizumab	Greece
NCT04335305	Recruiting	No results available	COVID-19	Drug: Tocilizumab biological: Pembrolizumab (MK-3475)	Spain
NCT04361552	Recruiting	No results available	COVID-19	Other: Best practice biological: Tocilizumab	United States
NCT04330638	Recruiting	No results available	COVID-19	Drug: Anakinra drug: Siltuximab drug: Tocilizumab	Belgium

COVID-19-specific convalescent plasma and immunoglobulin infusion

The use of convalescent plasma in severe acute respiratory infections (SARI) has shown favorable results in the past, with the mortality rate reduced by 75% in those who received convalescent plasma among all viral etiologies such as SARS coronavirus (SARS-CoV), Spanish influenza A (H1N1), Avian influenza A(H5N1), and influenza A (H1N1). These studies did not report any major adverse events [27]. COVID-19-specific plasma collected from five recovered SARS-COV2 patients with age ranges from 18 to 60, has shown positive results in a small case series of five critically ill patients diagnosed with COVID-19 who were under mechanical ventilation, and had a high viral load despite receiving antivirals and methylprednisolone. Among a total of five patients (age range: 36-73) who were treated with plasma, viral load reduction has been noted on the same day with continuous improvement over several days (1-12days) followed by gradual resolution of symptoms. Four out of five patients no longer required respiratory support on day nine post plasma transfusion and 3/5 patients were discharged home after hospital stay ranging from 51-55 days. Hospitalization was continued for 2/5 patients with a total of 37 days of hospital stay [28]. Similarly, the use of a high dose of intravenous immunoglobulin G (IVIG) obtained from healthy donors’ serum in patients (n = 3) with the diagnosis of COVID-19 severe type has also shown effective results. The fever subsided the very next day in two cases (age: 34 and 35) and the same day in the third patient (age 56), accompanied by symptoms resolution over a couple of days with no adverse events reported. This indicates that the clinical outcome of patients with COVID-19 can be improved if the disease progression is halted down by administering high dose IVIG in a timely fashion [29]. Additional data and clinical trials on high dose IVIG in COVID-19 patients are warranted. The ongoing clinical trials are given in Table 8.

**Table 8:** Characteristics of clinical trials studying specific convalescent plasma and immunoglobulin infusion in patients with COVID-19

Clinical trial ID	Status	Study results	Conditions	Interventions	Locations
NCT04358783	Recruiting	No results available	COVID-19	Biological: plasma Other: Best available therapy	Mexico
NCT04362176	Recruiting	No results available	COVID-19	Biological: pathogen reduced SARS-CoV-2 convalescent plasma biological: Placebo	United States
NCT04346589	Recruiting	No results available	COVID-19	Biological: Anti-coronavirus antibodies (immunoglobulins) obtained with double-filtration plasmapheresis from convalescent patients	Italy

Angiotensin receptor blockers and ibuprofen

Angiotensin-converting enzyme 2 (ACE2) is a binding site for SARS-CoV-2. The binding of SARS-CoV-2 to ACE2 leads to the downregulation of ACE2 resulting in increased angiotensin production by Angiotensin-converting enzyme (ACE) as there is less ACE2 to convert angiotensin to the vasodilator heptapeptide angiotensin 1-7. This increased angiotensin causes increased angiotensin receptor 1 (AT1R) stimulation resulting in increased pulmonary vascular permeability leading to lung injury. It has been hypothesized that AT1R antagonists like Losartan increase ACE2 expression and prevent lung injury in COVID-19 patients [30]. However, another hypothesis suggests that cellular entry of SARS-CoV-2 is facilitated by the up-regulation of ACE2 caused by AT1R antagonist use and hence can be harmful in COVID-19 patients. Similarly, non-steroidal anti-inflammatory drugs (NSAIDs) like Ibuprofen also acts by upregulating ACE2 [31]. Ongoing clinical trials are given in Table 9.

**Table 9:** Characteristics of clinical trials studying losartan in patients with COVID-19

Clinical trial ID	Status	Study results	Conditions	Interventions	Locations
NCT04330300	Recruiting	No results available	Hypertension, COVID-19	Drug: ACE inhibitor other antihypertensive agents	Ireland
NCT04340557	Recruiting	No results available	COVID-19	Losartan	United States
NCT04335786	Recruiting	No results available	COVID-19	Drug: Valsartan (diovan) drug: Placebo oral tablet	Netherlands
NCT04312009	Recruiting	No results available	COVID-19	Drug: Losartan other: Placebo	United States
NCT04311177	Recruiting	No results available	COVID-19	Drug: Losartan other: Placebo	United States
NCT04328012	Recruiting	No results available	COVID-19	Drug: Lopinavir/ritonavir Drug: Hydroxychloroquine Sulfate Drug: Losartan	United States

Antimalarial drug and its combinations

The 90% effective concentration (EC90) value of chloroquine against the SARS-CoV-2 in Vero E6 cells is 6.90 μM, which can be achieved clinically, as has already been demonstrated in the plasma of rheumatoid arthritis patients [32]. In an open-label non-randomized clinical trial of hydroxychloroquine (HCQ) (n = 36), 70% of HCQ treated patients (n=14/20) were virologically cured vs 12.5% in the control group (n=2/16) (*p* = 0.001) at day six post inclusion while 100% patients treated with HCQ plus Azithromycin (n = 6/6) were virologically cured vs 57.1% in patients receiving HCQ alone (*n* = 8/14) [32]. The limitations of this study were its small sample size, limited long-term outcome follow-up, and dropout of six patients from the study. Hence a larger sample size study is essential to investigate and find out the efficacy of HCQ in the treatment of COVID-19 [33]. However, in a recent study, hydroxychloroquine was not effective against COIVD-19 patients and has been revoked from the emergency use against COVID-19 patients due to safety concerns. Ongoing clinical trials are given in Table 10.

**Table 10:** Characteristics of clinical trials studying chloroquine and hydroxychloroquine in patients with COVID-19

Clinical trial ID	Status	Study results	Conditions	Interventions	Locations
NCT04316377	Recruiting	No results available	COVID-19	Drug: Hydroxychloroquine sulfate	Norway
NCT04341727	Recruiting	No results available	COVID-19	Drug: Hydroxychloroquine sulfate drug: Azithromycin Drug: Chloroquine sulfate	United States
NCT04346667	Recruiting	No results available	COVID-19	Drug: Hydroxychloroquine sulfate Regular dose. Drug: Hydroxychloroquine sulfate loading dose. Drug: Chloroquine	Pakistan
NCT04322396	Recruiting	No results available	COVID-19	Drug: Azithromycin drug: Hydroxychloroquine	Denmark
NCT04351191	Recruiting	No results available	COVID-19	Drug: Hydroxychloroquine sulfate regular dose. Drug: Hydroxychloroquine sulfate loading dose	Pakistan
NCT04325893	Recruiting	No results available	COVID-19	Drug: Hydroxychloroquine drug: Placebo	France
NCT04353037	Recruiting	No results available	COVID-19	Drug: Group a hydroxychloroquine drug: Group b control	United States
NCT04324463	Recruiting	No results available	COVID-19	Drug: Azithromycin drug: Hydroxychloroquine or chloroquine	Canada
NCT04321278	Recruiting	No results available	COVID-19	Drug: Hydroxychloroquine + azithromycin drug: Hydroxychloroquine	Brazil
NCT04344951	Recruiting	No results available	COVID-19	Drug: Chloroquine phosphate 200mg tablets	Greece
NCT04342221	Recruiting	No results available	COVID-19	Drug: Hydroxychloroquine sulfate drug: placebo	Germany
NCT04351724	Recruiting	No results available	COVID-19	Drug: Chloroquine or hydroxychloroquine drug: Lopinavir/ritonavir	Austria

## Conclusions

Collaborative global efforts are underway to control the COVID-19 pandemic. Many clinical trials are underway. These trials are different in study structure, the severity of the disease, the duration of the treatment, and the dosing of drugs in the target population. These trials are also different in the quality of the reported information. Many studies are going in parallel, suggesting that the scientific community is working hard to come up with an effective treatment for COVID-19. Few drugs like remdesivir and dexamethasone have demonstrated positive early results against COVID-19 in clinical trials and their efficacy has also been observed in pre-clinical and clinical studies, but their use against COVID-19 should be based on the severity of patient clinical status and ethical approval from randomized clinical trials. Efficacy and safety data from further studies with larger sample size, blinded, randomized design, and longer follow-up are needed urgently.
